# Transferring the approach avoidance task into virtual reality: a study in patients with alcohol use disorder versus healthy controls

**DOI:** 10.1007/s10055-023-00835-7

**Published:** 2023-08-03

**Authors:** Leonie Ascone, Janina Wirtz, Angelina Isabella Mellentin, Dimitrij Kugler, Thomas Bremer, Friedrich Schadow, Stine Hoppe, Charlotte Jebens, Simone Kühn

**Affiliations:** 1 Neuronal Plasticity Working Group, Department of Psychiatry and Psychotherapy, University Medical Center Hamburg-Eppendorf, Martinistr. 52, 20246 Hamburg, Germany; 2 Lise Meitner Group for Environmental Neuroscience, Max Planck Institute for Human Development, Lentzeallee 94, 14195 Berlin, Germany; 3 Unit for Clinical Alcohol Research, Unit for Psychiatric Research, Department of Clinical Research, University of Southern Denmark, J. B. Winsløwvej 18, 5000 Odense C, Denmark; 4 Brain Research-Inter-Disciplinary Guided Excellence (BRIDGE), Department of Clinical Research, University of Southern Denmark, 5000 Odense C, Denmark; 5 Center for Digital Psychiatry (CEDIP), Heden 11, 5000 Odense C, Region of Southern Denmark Denmark; 6 University of Applied Sciences, Treskowallee 8, 10318 Berlin, Germany; 7 Department of Clinical Psychology and Psychotherapy, Christian-Albrechts-University Kiel, Christian-Albrechts-Platz 4, 24118 Kiel, Germany; 8 Max Planck-UCL Center for Computational Psychiatry and Ageing Research, Max Planck Institute for Human Development, Lentzeallee 94, 14195 Berlin, Germany

**Keywords:** Approach bias, AAT, Alcohol use disorder, Virtual reality, Validation

## Abstract

Study aims were to (I) transfer the measurement of the approach bias (Apb) related to alcoholic stimuli via the Approach Avoidance Task (AAT) into Virtual Reality (VR), (II) check whether measuring Apb in VR leads to similar or different results compared to the classical PC-based version, (III) check the validity of VR versus PC-based bias scores in terms of relatedness to clinical variables. Different ‘grasping-conditions’ were tested and contrasted in VR concerning (Ia) feasibility (performance): (1) *never grasp*, (2) *always grasp*, (3) *grasp* when *PULLing* stimuli towards oneself. (Ib) Differences in the bias scores between patients with alcohol use disorder (AUD) and healthy controls (HC) were examined for each grasping-condition. (II) PC-based bias scores were computed and contrasted for AUD versus HC. (III) Correlations of the different VR- versus PC-based bias scores with AUD symptom severity and impulsivity were checked to evaluate validity. (Ia) Grasping-condition 1, followed by 3, showed acceptable (> 50%) and good (> 80%) rates of correct performances allowing for robust median estimation. (Ib) Significant differences in the resulting bias scores emerged between AUD and HC only for grasping-condition 1 (*p* = 0.034) and 3 at trend-level (*p* = 0.093). For grasping-condition 1 the Apb Median for AUD was different from zero at a non-significant trend-level (*p* = 0.064). (II) The PC-based bias scores did not discriminate between AUD versus HC groups. (III) Grasping-condition 1 and 3 VR-based bias scores correlated significantly with impulsivity. In sum, transferring the AAT into VR is feasible, valid, and best implemented without an additional grasping-component when using the VR-controller. This way of Apb assessment represents a viable, perhaps even superior, alternative to PC-based assessments.

*Trial registration* The trial was pre-registered at AsPredicted #76854: ‘Transferring the approach avoidance task into virtual reality’, 10/13/2021; prior to any analyses being undertaken.

## Introduction

Overconsumption of alcohol poses a worldwide risk with 3 million deaths per year and 283 million people who suffered from alcohol use disorder (AUD) in 2016 (WHO 2018). AUD treatment typically includes pharmacotherapy, psychotherapy, and behavioural interventions. Unfortunately, despite the integration of a variety of treatment approaches, relapses after the end of treatment (especially in the first year) are common (Litten et al. 2015). Thus, the understanding and measurement of patho-mechanisms are crucial. The classical psychological dual-process model proposes two different, competing neuro-behavioural mechanisms or pathways: (1) a fast, unconscious *impulsive system/pathway* versus (2) a conscious *reflective system/pathway*. Transferring this concept to AUD, several assumptions, delineated from the dual-process model can be made and put to the test. First, a general predominance of the impulsive system is assumed, along with decreased reflective system function and/or capacity. Second, more specific assumptions concerning aberrant neuronal and thus behavioural reactions to alcoholic cues in AUD result from the notion of the general predominance of the impulsive system. Mainly, three systems can be broadly summarized that respond aberrantly to alcoholic cues in individuals with AUD (sorted from early to later processing/ response stages): the attentional system (*attentional biases*), reward system (*cue reactivity, craving*), and motor system (*automated approach behaviour*). Indeed, research confirms that in addictive disorders there is a predominance of the automatic system, for instance, evidenced by the tendency to direct attention towards, and approach, craved stimuli compared to neutral stimuli (Wiers et al. 2010; Fridland et al. 2017; Wiers et al. 2013a; Wiers et al. 2013b; Watson et al. 2012).

A treatment relying specifically on the notion of motoric automated approach biases (Apbs) towards alcohol that has shown preliminary promise in clinical settings is the approach-avoidance training programme (*AATP*), which builds on the experimental alcohol-approach-avoidance task (alcohol *AAT*) (Rinck and Becker 2007; Wiers et al. 2009), which measures Apbs. Hence, the AAT can be both utilized as an experimental tool to detect such approach tendencies, as well as to re-train them, in a variety of addictive disorders (Kakoschke et al. 2017) including AUD (Wiers et al. 2010, 2011). It is important to point out that the AAT cannot be utilized to diagnose clinical disorders such as AUD, yet the experimental version of the AAT has oftentimes, though not continuously, allowed for the detection of approach biases in the conditions mentioned above. The AAT and AATP are usually conducted on a computer, where participants are confronted with pictures of craved alcoholic stimuli versus neutral objects on the computer screen. In the so-called *explicit* version of the AAT, they are instructed to directly approach the alcoholic stimuli versus avoid the neutral control stimuli in one block, and to do the opposite in the consecutive block (i.e. avoid alcoholic stimuli and approach neutral ones), by pushing (= avoidance movement) or pulling (= approach movement) a joystick. The reaction times (RTs) often show that craved (alcoholic) objects are approached much faster than neutral ones by individuals with addiction or risky consumption, indicating an Apb, (Kakoschke et al. 2017; Wiers et al. 2010, 2011).

The final goal of the training (AATP), which follows the experimental AAT, is to retrain automatic approach tendencies. Here, participants are trained to always push alcoholic stimuli away as fast as possible to counteract the existing approach tendency. As may have become apparent by this summary, a valid and accurate measurement of the Apb is crucial both from a theoretical (concerning the mechanisms) and clinical point of view. However, critically, an Apb towards alcohol could not always be replicated throughout the literature (Barkby et al. 2012; Spruyt et al. 2013). One reason may be the method of assessment on a PC, presenting objects on a screen, and using a joystick for the movements, which is quite abstract and far off from real-life situations. The utilization of a joystick to carry out the push and pull movements in the classical PC-based AAT is a rather sparse motion, albeit a fundamental concept of the AAT is embodiment—the notion that motions, emotions and cognitions are inter-connected and occur automatically (Dijkstra et al. 2015; Fridland et al. 2017; Dijksterhuis et al. 2001). A virtual reality (VR) environment could be ideally suited to overcome these problems. The VR could be used to enhance both ecological validity via immersion in a virtual environment during the experimental AAT, and embodiment via more realistic arm movements and hand-to-object interactions.

It is thus of central interest for the present study, whether the Apb might be easier to detect in a VR setting (VR-AAT) than using the classical computer-joystick-setting (PC-AAT). Results of two studies from the same research group investigating the AAT concept in a virtual environment (Kim and Lee 2015, 2019) showed that the AATP might decrease approach tendencies towards alcohol in a sample with subthreshold AUD (heavy social drinkers; Kim and Lee 2019). However, an experimental VR-AAT to quantify the Apb has not been formally developed and compared directly to the original experimental PC-AAT yet. Especially from the background that a VR-AATP could represent a beneficial treatment add-on for patients with AUD, it is of interest to investigate whether implementing the experimental AAT in a virtual environment (I) is feasible, (II) enhances the detection of Apbs, and (III) is valid (i.e. assessed bias scores correlate with clinical criteria).

### The present study

The present study was dedicated to investigating three major research questions. (I) Could mimicking the ‘grasping’ of stimuli in an explicit VR-version of the AAT enhance embodiment, as reflected in more marked expressions of the Apbs in patients with AUD compared to healthy controls (HCs)? To test this, three different ‘grasping-conditions’ were implemented: (1) never use (press) the lever at the underside of the VR-controller (‘*never grasp’*), (2) ‘*always grasp’* the stimuli by pressing the lever at the underside of the VR-controller, both when PUSHing stimuli away or PULLing them towards oneself, (3) ‘grasp’ the stimuli only when PULLing them towards oneself, but not when PUSHing them away (‘*grasp PULLing*’). The ‘grasping-conditions’ were examined concerning (Ia) feasibility (i.e. if participants were able to perform the movements correctly within a given number of trials) and (Ib) which of the conditions show the clearest difference in the Apb when comparing AUD patients to HCs. (II) Are the results concerning the Apb in VR equivalent to or different from those obtained using a classical explicit PC-based AAT version? Again, whether AUD patients and HC differed significantly with respect to the expressions of the bias scores was assessed. (III) Which of the alternative measures of the bias scores (VR-based vs. PC-based Apbs) correlate significantly with clinical variables (i.e. severity of AUD, impulsivity)? Thereby, it was examined whether there was no, similar, or differential evidence for convergent validity concerning VR-based versus PC-based versions of the AAT.

## Methods

### Recruitment, in- and exclusion criteria

The trial was pre-registered (10/24/2020) at aspredicted.org (AsPredicted #76854: ‘Transferring the approach avoidance task into virtual reality'). To focus on the methodology described in this paper and to simplify reporting, only assessments relevant to this study will be described. In all procedures, we adhered to the declaration of Helsinki and the study was approved by a local ethics board prior to study onset (LPEK-0088). We obtained informed consent from all participants who were enrolled in the study. Patients with AUD were recruited from the patient ward for addictive disorders at the University Medical Center Hamburg-Eppendorf. HCs were recruited via local and online advertisements. Participants in general had to be ≥ 18 years of age, right-handed or ambidextrous (due to how the VR was programmed).

The main inclusion criterion for the AUD group was a current AUD diagnosis (hospital case file). The diagnosis was verified using the MINI International Neuropsychiatric Interview German Version 5.0.0 (Sheehan et al. 1998). Furthermore, patients were only included if they had no current withdrawal syndrome (i.e. completed detoxification program), no comorbid addiction (with the exception of tobacco), no current anticonvulsive or neuroleptic treatment, no severe psychiatric comorbidity (e.g. psychosis, bipolar disorder), no neurological disorder, no impaired visuo-motor skills, no language-related deficits or impairments (e.g. aphasia), sufficient command of the German language, and no severe somatic disorders. Potential HCs underwent an online pre-screening via the platform *LimeSurvey* (https://www.limesurvey.org/), whereby it was assessed whether they had any past or present psychiatric disorders (as later further verified with the MINI in the lab). In addition, they were only invited to participate if they scored < 8 on the Alcohol Use Disorder Identification Test (AUDIT; Babor et al. 2001), to exclude risky drinking behaviour and to assure a marked contrast to the clinical group. All further exclusion criteria were the same as for the patient group and were checked during pre-screening.

### Procedure

**Fig. 1:**
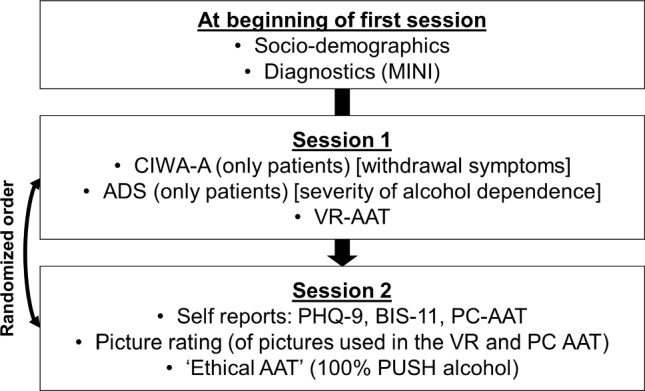
Experimental procedure. *Note*. *CIWA-A* Clinical Institute Withdrawal Assessment for Alcohol Scale, *ADS* alcohol dependence scale, *VR-AAT* approach avoidance task in virtual reality, *PC AAT* approach avoidance task on PC, *PHQ-9* patient health questionnaire-9, *BIS-11* behavioural inhibition scale

The participants were invited to two separate testing sessions (A vs. B; see Fig. 1) on two subsequent days. Each session lasted 1–2 h and started at a similar time of the day. Diagnostics were always conducted at the beginning of the first session to clarify (or exclude) existing psychopathology in general and severity of AUD specifically. Additional questionnaires assessed depression (PHQ-9; Kroenke et al. 2001) and impulsiveness (BIS-11; Patton et al. 1995).

It was randomized in which order, hence on which day, participants completed the VR-AAT (session A) versus conventional PC-AAT (session B). As a previous study showed that Apbs were larger when stimuli were task-relevant (Lender et al. 2018), the explicit version of the experimental AAT was utilized for both PC- and VR-AAT in this study. At the end of the second day of the study, all participants carried out an ‘ethical AAT’ on the computer (identical amount of trials as the other AATs), in which they explicitly and consistently pushed alcohol away from themselves. This was implemented to ensure participants, and especially AUD patients, did not leave the lab after being instructed to pull alcoholic stimuli towards themselves, possibly leading to heightened craving, putting them at heightened risk for consumption. Further experimental details are provided in the following sections.

### Experiment

**Fig. 2:**
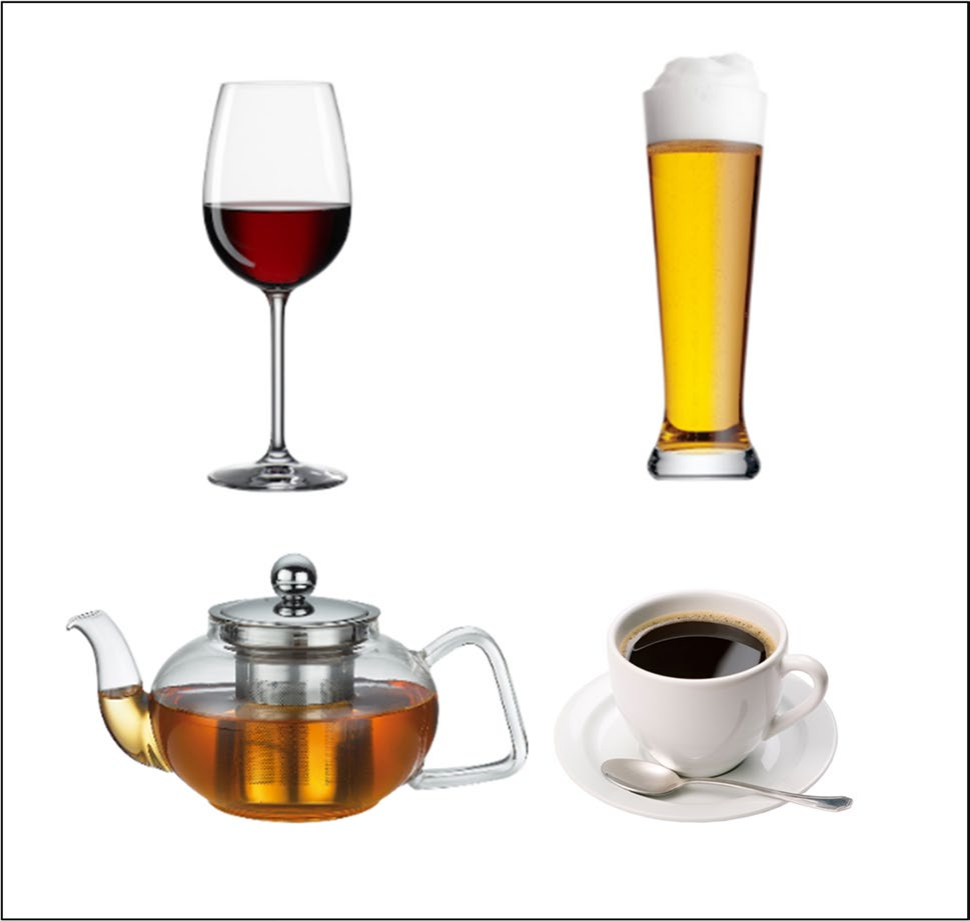
Exemplary AAT stimuli as used in the VR- and PC-AAT versions. *Note*. For reasons of trademark protection, only non-brand stimuli are shown in this figure

*Stimuli* The same 39 neutral and 39 alcoholic stimuli (images of beverages) were used both for the VR- and PC-AAT. These were selected from a larger picture set of beverages from our lab (Kugler et al. submitted) and matched based on visual judgement concerning proportion, lighting, and perspective (for exemplary pictures from the set, see Fig. 2. Each were photographs of the respective beverage on a white background (image resolution 2100 × 1500 px). The selection of the stimuli was also driven by covering different types of alcohol as well as brands commonly available in Germany. Furthermore, the stimulus set was varied in that different types of beverages were presented brand-free, i.e. in glasses or other representative vessels (e.g. pints, mugs, pots, cocktail/wine glasses, etc.).

**Fig. 3:**
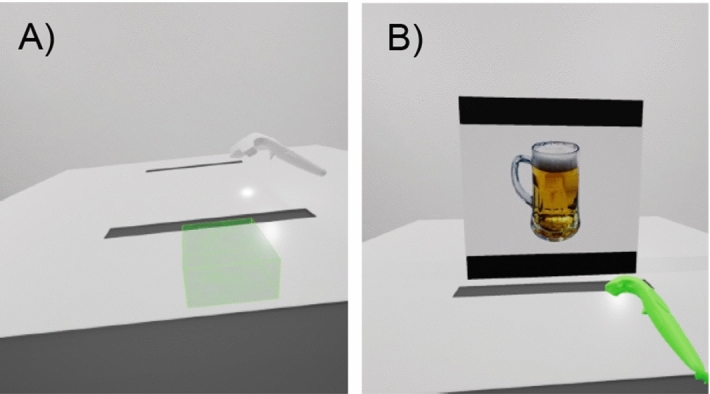
Screenshots from the VR-AAT setting. Note. **A** Shows the table with the two slots for sorting in the pictures, as well as the marker cube (in green) in which participants had to keep their hand (holding the controller) until the stimulus appeared; **B** exemplary trial, pulling an alcoholic beverage into the according slot.

*VR-AAT implementation* To assure comparability between the PC and VR versions, except for the movement- and hand-object-interaction factor, other experimental parameters were kept simple. For instance, participants were seated at a table with the same height as the computer desk of the PC-AAT. Instead of creating 3D objects, 2D picture stimuli were used in VR, matching the presentation in the PC-AAT version (see Fig. 3). In block (A) participants were explicitly instructed to PUSH alcohol away from themselves (PUSH_alc_) while PULLing non-alcoholic beverages towards themselves (PULL_soft_) versus block (B) to PULL alcohol towards themselves (PULL_alc_) while PUSHing non-alcoholic beverages away (PUSH_soft_). Participants were wearing a HTC VIVE Pro HMD VR Headset (2880 × 1600 pixels) and used the appertaining VR-controller to reach out to the stimulus, appearing in front of them in the centre of a white table, which they then had to correctly sort into a slot close to them (PULL) versus at the other side of the table (PUSH).

The controller had to be held within a ‘marker cube’, placed centrally at the edge of the table closest to the participant, until the stimulus appeared (onset of RT measurement). This was done to assure standardized measurements of RTs concerning starting point and distance. Upon the stimulus’ appearance, participants had to either PUSH the stimulus away by quickly extending their arm forward hitting the stimulus versus PULLing it towards themselves as quickly as possible. The PULLing movement had to be conducted as if grasping the beverage slightly from the side. This distinction in initial hand-to-object-contact (hit vs. grasp) technically enabled the tracking system and software to distinguish the movements. Matching the PC-AAT version, each stimulus was shown once in each block, resulting in 78 trials per block (39 alcoholic, 39 non-alcoholic stimuli; see Stimulus subsection for details). Before starting each block, as many practice trials as needed were run for the participant to train the respective movement and grasping-condition with two picture stimuli not used in the main blocks (a glass of beer vs. a glass of water).

As we were particularly interested in whether adding a grasping-component could enhance ‘embodiment’ and accordingly the manifestation of the VR-based Apb, several grasping-conditions, utilizing the lever at the underside of the controller were implemented: (1) never press the lever interacting with the stimuli (never grasp); (2) always press the lever to ‘grasp’ a beverage regardless of whether PUSHing or PULLing any of the stimuli (always grasp); (3) use the lever to ‘grasp’ stimuli only when PULLing them towards the self (grasp PULLing). This resulted in a 2 (order of explicit instructions A–B vs. B–A) × 3 (grasping-conditions) within-subject design with 6 blocks per subject, while randomizing both the order of the explicit instruction and the order of the grasping-conditions. An overview of the resulting conditions is given in Table 1*.*

**Table 1:** Overview of all VR-AAT blocks and conditions

Grasping- condition		Explicit instruction concerning stimulus type	Grasping-instruction
1	A	PUSH_alc_ (39 trials)−PULL_soft_ (39 trials)	Never use the lever (*never ‘grasp’*)
	B	PULL_alc_ (39 trials)−PUSH_soft_ (39 trials)	
2	A	PUSH_alc_ (39 trials)−PULL_soft_ (39 trials)	Always press the lever to ‘grasp’ beverage (*always ‘grasp’*)
	B	PULL_alc_ (39 trials)−PUSH_soft_ (39 trials)	
3	A	PUSH_alc_ (39 trials)−PULL_soft_ (39 trials)	Press the lever only when PULLing stimuli towards the self (‘*grasp’ PULLing*)
	B	PULL_alc_ (39 trials)−PUSH_soft_ (39 trials)	

*The order of grasping-conditions (1–3) was randomized, and the order of the explicit instructions was randomized within grasping-condition-blocks

*VR-AAT detailed reaction time and bias score computation approach* There is presumably a difference in the variability of the PULL versus PUSH RTs in VR due to an enhanced cognitive load in the PULL conditions. In the PULL conditions (grasping-conditions 2 & 3), participants needed to bear in mind pressing the lever when performing the movement and in addition the PULL movement needed always to be conducted slightly from the side, hence distinguishing it from the PUSH movement. Thus, when computing the Apb for alcoholic stimuli, the assumed greater potential variability in RTs between PUSH and PULL conditions should be considered by separately standardizing the respective median RTs. Positive values indicate an Apb towards alcoholic beverages; negative values accordingly indicate an avoidance bias.

The complete RT (RT_total_), i.e. the timespan between the stimulus appearing and the endpoint of the movement to react to it by correctly sorting the stimulus into one of the slots (situated at the end of the table for PUSH and in front of oneself for PULL) without changing direction (i.e. correcting the initial reaction), was inspected. All individual RTs for all subjects were screened using an automatic script to detect outliers. The outliers mostly represented excessively prolonged reaction times due to technical difficulties with the VR gear, or issues handling the VR controller and were defined as exceeding ± 2 SD boundaries around the total sample median. This was established separately for PUSH_alc_ versus PULL_alc_ RT_total_ (see Results section for statistics).

In addition, medians for a respective subject and condition were only computed if at least 25 RT_total_ per condition were available (1A, 1B, 2A, 2B, 3A, 3B; see Table 3). This rule translates to a ratio of 25/39 trials, ≈ 64% of data available to compute the respective median. This constraint was defined as due to the enhanced complexity of movements in the VR, some participants did not perform them correctly. This included prematurely leaving the marker cube before onset of the stimulus, extreme latencies, incorrectly sorted stimuli, change of direction, or largely untraceable movements (i.e. movement tracking was impossible due to gross deviations from the instructed movement). Hence, individual participants could have large amounts of ‘invalid’ data not recorded by the programme. Based on the ‘25 RT_total_ per condition’ rule of thumb, robustness of the estimated medians was ensured. The evaluation of the number of participants with over versus below 64% of data for each of the 6 conditions also allows for an evaluation of feasibility concerning the technical aspects of the VR and was regarded as an additional descriptive outcome.

### PC-AAT implementation

Like the VR-AAT, the PC-AAT was run in an explicit version with two blocks, whereby participants were instructed to (A) PUSH_alc_−PULL_soft_ versus (B) PULL_alc_−PUSH_soft_. The order of these instructions (and according blocks of trials) was randomized. A joystick (Speedlink; model: *Dark Tornado Flight Stick*) was used to implement the movements. Visual feedback was provided by zooming the stimulus in (PULL) or out (PUSH), enlarging versus shrinking the stimuli, respectively, such as to mimic it approaching the participant versus moving away.

As in the VR-AAT, each stimulus was shown once in each block, resulting in 78 trials per block (39 alcoholic, 39 non-alcoholic stimuli; see Stimulus subsection). Before starting each block, 10 practice trials were run with 2 picture stimuli only, that were not used in the main trials (same as in the VR-version: glass of water and a glass of beer). The programme was run using the software *Inquisit 4* (Millisecond), using the beta version (Borchert 2014) of the AAT as provided on the website. No more than 3 stimuli of the same category (alcohol vs. no alcohol) were presented successively.

### PC-AAT detailed reaction time and bias score computation approach

The outcome of interest was the Apb score, defined exactly as for the VR-based bias score: with the Apb score measured with the PC-AAT version being defined by subtracting the median reaction time (Median_RT) for PULLing alcoholic stimuli (alc) towards oneself (hypothesized to be especially fast in patients with AUD) from the Median_RT for PUSHing alcoholic stimuli away. The median RTs in the numerator need to be standardized by the variability of RTs for both conditions (PUSHing vs. PULLing alcoholic beverages) in the denominator, which is defined by the respective standard deviations (SDs). Positive values indicate an average Apb towards alcoholic beverages, while negative values indicate an avoidance bias.

The total RT, i.e. the timespan between the stimulus appearing and the endpoint of the movement to correctly react to it by fully extending the joystick in either direction (PUSH or PULL), without changing direction (i.e. revising the initial reaction), was inspected (RT_total_). All individual RTs, for all subjects, were screened using an automatic script to detect outliers. Outliers were defined as exceeding ± 2 SD boundaries around the total sample median, hence establishing a 95% confidence interval (CI see Results section for statistics).

### Measures

*Diagnostic measures for general psychopathology and severity of AUD* The Mini-International Neuropsychiatric Interview German Version 5.0.0 (MINI; Sheehan et al. 1998) is a semi-structured interview based on the Diagnostic and Statistical Manual of Mental Disorders (DSM-5). It is used to verify a diagnosis of AUD by assessing the severity of alcohol dependency (0 = none-minimal, 1 = mild, 2 = moderate, 3 = moderate-severe, 4 = severe) and to screen for comorbid mental disorders.

The Alcohol Use Disorders Identification Test (AUDIT; Babor et al. 2001) contains 10 items and measures the severity of alcohol-related problems. It served as a screening tool to exclude HC with a score of 8 or higher, which indicates risky drinking behaviour.

The Alcohol Dependence Scale (ADS; Skinner et al. 1984) assesses both the presence and severity of AUD. It is comprised of 25 items to rate the presence of AUD-related problems and symptoms either dichotomously (i.e. yes vs. no), or on a 3- or 4-point scale (e.g. quantifying different intensities). The total scores can range between 0 and 47. A low level of alcohol dependence is indicated by a scores < 14, an intermediate level by a score between 14 and 21, a substantial level by a score between 22 and 30, and a severe level by a score > 31.

The Clinical Institute Withdrawal Assessment for Alcohol (CIWA-Ar; Sullivan 1989) is a 10-item scale assessing clinical quantitation of alcohol withdrawal symptoms severity. The items are rated on 4- to 7-point scales, and the maximum score is 67. No to minimal withdrawal symptoms are indicated by a score of 0 to 9, mild to moderate withdrawal symptoms by a score of 10 to 19, and severe withdrawal symptoms by a score > 20.

*Additional questionnaires* The Patient Health Questionnaire (PHQ-9; Kroenke et al. 2001) assesses depression on a 4-point Likert scale (0 = not at all, 1 = on some days, 2 = on more than half of the days and 3 = almost every day) and captures the nine criteria for depression according to the DSM-IV. Scores < 5 indicate no depression, scores < 10 are evaluated as unobtrusive, while scores between 10 and 14 indicate mild, 15–19 medium, and 20–27 severe depressive symptoms.

Impulsivity was assessed with the 30-item Barratt Impulsiveness Scale (BIS-11; Patton et al. 1995). It focuses on three dimensions of impulsivity: (1) attentional subscale (attention and cognitive instability), (2) motor subscale (motor and perseverance), and (3) non-planning subscale (self-control and cognitive complexity), where items are rated on a 4-point Likert scale (1 = rarely/never, 2 = occasionally, 3 = often, and 4 = almost always/always).

## Results

### Sample

The total sample (*N* = 57) consisted of 25 AUD patients and 32 HCs. The participants had a mean age of 46.86 years, and 54% were male. The two groups did not differ significantly with respect to age, sex, school education and VR rating (see Table 2).

**Table 2:** Sample characteristics and group differences for AUD versus HC group. Frequencies (%) or means (SD) for demographic, treatment-related, and psychopathological parameters (*N* = 57)

	Total (*N* = 57)	AUD (*n* = 25)	HC (*n* = 32)	Inferential statistics
Age mean (SD) in years	46.86 (13.87)	48.28 (12.64)	45.75 (14.91)	*t*(55) = 0.68, *p* = 0.250
Sex—males (%)	30 (53.57%)	14 (58.33%)	16 (50.00%)	*X*^*2*^(1, *N* = 56) = 0.38, *p* = 0.536
School education (categories 0–3)^1^	1/14/20/22	1/7/9/8	0/7/11/14	*X*^*2*^(3, *N* = 57) = 2.01, *p* = 0.571
PHQ-9	7.88 (7.03)	12.36 (7.84)	4.26 (3.34)	*t*(54) = 5.21,* p* < 0.001*
*BIS-11*	61.25 (11.31)	65.48 (11.53)	57.94 (10.12)	*t*(55) = 2.26,* p* = 0.011*
*CIWA-A*	1.04 (1.97)	1.04 (1.97)	*–*	*–*
*AUDIT mean (SD)*	1.96 (1.91)	*–*	1.96 (1.91)	*–*
ADS mean (SD)	21.40 (8.03)	21.40 (8.03)	–	*–*
Number of MINI diagnoses	1.46 (2.24)	3.28 (2.34)	0.03 (0.18)	*t*(55) = 7.86, *p* < 0.001*
Severity of alcohol dependency according to Mini	26/12/10/2/6	7/3/7/2/6	19/9/3/0/0	*X*^*2*^(5, *N* = 57) = 18.56, *p* = 0.002*
VR rating	1.77 (0.78)	1.68 (0.69)	1.84 (0.85)	*t*(55) =—0.78, *p* = 0.436

^1^AUD = alcohol use disorder; HC = healthy controls; categories according to German school system; 0 = no school degree; 1 = low school degree; 2 = middle school degree; 3 = highest school degree. Data in absolute numbers per category and percentage within each group and category. PHQ-9 score interpretation: < 5 = healthy, < 10 unobtrusive, 10–14 mild depressive symptoms, 15–19 medium depressive symptoms, 20–27 severe depressive symptoms; BIS-11 score interpretation: 1 = rarely/never, 2 = occasionally, 3 = often, and 4 = almost always/always;CIWA-A score interpretation: score of < 8 = mild-, > 15 = severe withdrawal symptoms; AUDIT score interpretation: score ≥ 8 indicates risky drinking behaviour; ADS score interpretation: scores < 14 = low level, 14- 21 = intermediate level, 22–30 = substantial level and > 31 = severe level of alcohol dependence; Severity of alcohol dependency according to Mini interpretation: none-minimal/ mild/ moderate/ moderate-severe/ severe

Validating the clinical versus healthy sample, AUD patients scored significantly higher on the PHQ-9 and BIS-11 scale (*p* < 0.001 and *p* = 0.011, respectively) compared to HCs. Furthermore, AUD patients presented with significantly more (comorbid) diagnoses, as assessed by the MINI (*M* = 3.28) than HC (*M* = 0.03; *p* < 0.001) and a significantly higher severity of alcohol dependency than HC on the MINI dependency severity scale (*p* = 0.002).

AUD patients displayed an intermediate level of alcohol dependence, as measured by the ADS (*M* = 21.40) with scores ranging from 4 to 33. However, withdrawal symptoms, measured using the CIWA-A were only mild (*M* = 1.04), with a minimum score of 0 and a maximum score of 8. They also exhibited higher scores on impulsivity, as measured by the BIS-11 (*M* = 61.25), with a minimum score of 41 and a maximum score of 87. Lastly, AUD patients exhibited mild depressive symptoms, as measured by the PHQ-9 (*M* = 7.88; scores range between 0 and 26).

### Descriptive VR-AAT data analysis

The median RT_total_ of the raw data including all available RTs (i.e. correctly sorted, no change of direction, alcoholic stimuli; *N* = 10,225) for PUSH_alc_ (*n* = 5689 trials available) was Md_RT_total_PUSHalc_VR_ = 1344 ms (SD = 678 ms). This leads to a 2-SD upper bound of 2700 ms. For PULLing alcoholic stimuli, based on all available RTs (*n* = 4536 trials available) results were Md_RT_total_PULLalc_VR_ = 1590 (SD = 886 ms), corresponding to an upper bound of 3362 ms. Using these upper bounds as filters to sort out too long RTs, this resulted in a filtered dataset of *N* = 9834 (− 9.6%), with now *n* = 5473 (− 9.6%) available PUSH_alc_ and *n* = 4361 available PULL_alc_ (− 9.6%) RTs_total_ responses.

### (Ia) Feasibility of the different grasping-conditions in VR

In a next step, the different grasping-conditions (1–3) were evaluated per participant concerning the availability of a sufficient number of RTs (*N* = 25) to compute the respective median scores and standard deviations.

As shown in Table 3, some of the conditions seem to have been difficult to execute for most participants. Within grasping-condition 1 (*never grasp*), only 52% of AUD patients and 58% of HCs had ≥ 25 valid, paired PUSH & PULL responses. Within grasping-condition 2 (*always grasp*), the numbers were even lower with 12% and 13%, for AUD patients and HCs, respectively. Only in grasping-condition 3 (*grasp PULLing*), a reasonable number of participants (88% of AUD patients and 90% of HCs) reached a minimum of ≥ 25 valid, paired PUSH & PULL responses, suggesting this condition was best feasible to execute. In general, the low available sample sizes require a non-parametric inferential data analysis approach. The number of available cases in grasping-condition 2 was too low even for a non-parametric statistical approach; hence, no further results will be reported for this condition.

**Table 3:** Sample sizes available for each grasping-condition based on robust median estimation

	AUD			HC		
	*n*^1^%	PULL_alc_ (Median, SD)	PUSH_alc_ (Median, SD)	*n*^1^	PULL_alc_ (Median, SD)	PUSH_alc_ (Median, SD)
TOTAL	24 (96%)	1514 (252)	1430 (166)	31 (100%)	1630 (298)	1475 (263)
1—Never grasp (A&B)	13 (52%)	1610 (337)	1270 (198)	18 (58%)	1740 (339)	1395 (307)
2—Always grasp (A&B)	3 (12%)	1280 (155)	1085 (162)	4 (13%)	1640 (155)	1330 (030)
3—Grasp pulling (A&B)	22 (88%)	1471 (208)	1329 (189)	28 (90%)	1513 (258)	1295 (237)

AUD = alcohol use disorder; HC = healthy controls

^1^Refers to the available sample size (total participants within clinical vs. control group) with at least 25 valid RTs for PULL–PUSH alcohol, per condition

### (Ib) Inferential VR-AAT data analysis: group differences in the VR-approach biases

For grasping-condition 1 (*never grasp*), there was a significant difference in bias scores (Apb_VR_no_grasp_) between AUD patients and HCs, *U*(*N*_AUD_ = 13, *N*_HC_ = 18) = 64.0, *z* = 2.12, *p* = 0.034, with AUD patients descriptively showing an Apb and HCs showing an avoidance bias on average (Median_AUD_ = 1.167, SD = 1.873; Median_HC_ = − 0.623, SD = 1.819). The effect size of the difference between the groups is *η*^*2*^ = 0.15.

**Fig. 4:**
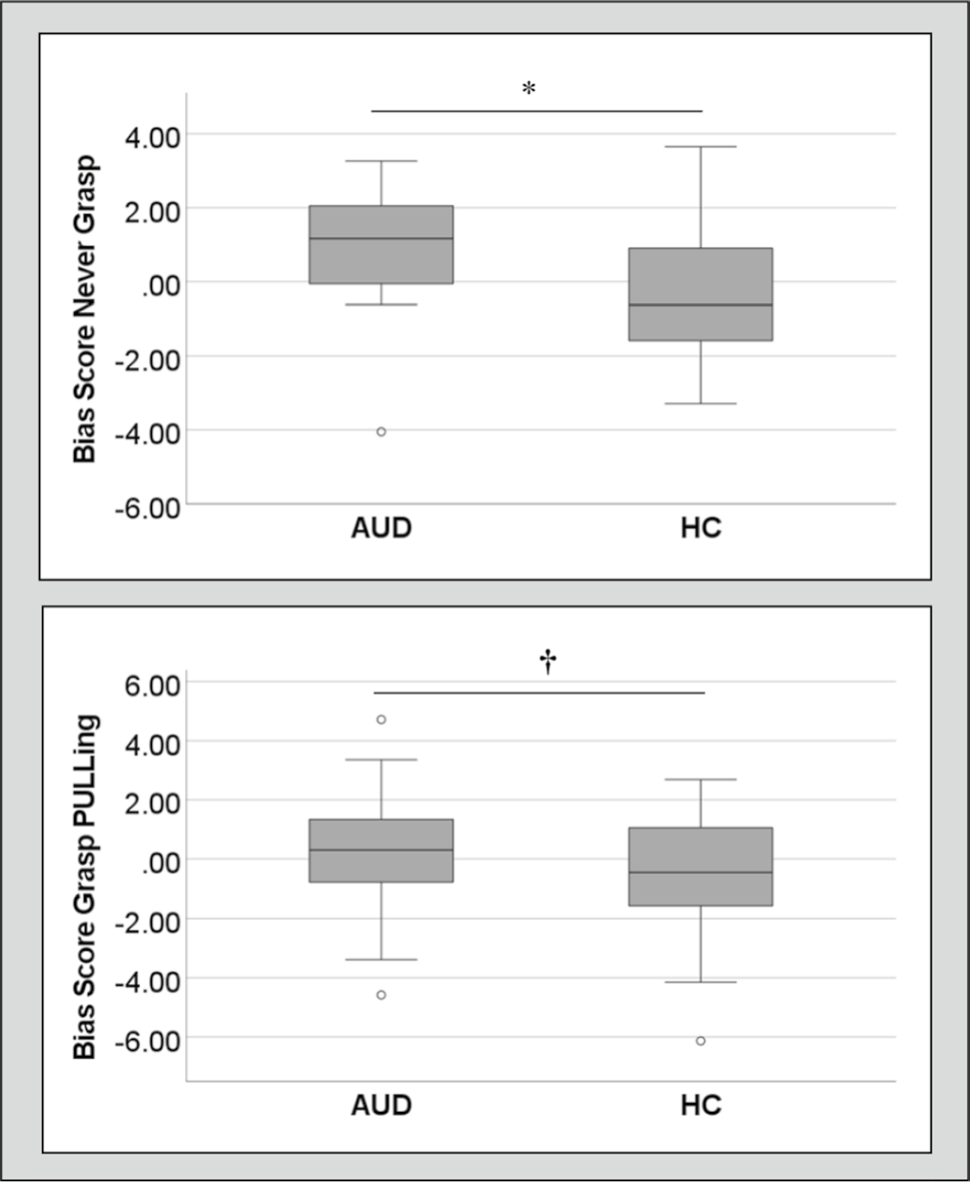
Boxplots for the different VR-AAT bias scores. *Note*. Circles denote outliers, which were not removed for analysis; *p < 0.05, †*p* < 0.10; AUD = alcohol use disorder; HC = healthy controls

For grasping-condition 3 (*grasp PULLing*), there was a non-significant trend-level difference in the bias scores (Apb__VR_grasp_PULLing_) between AUD patients and HCs, U(*N*_AUD_ = 22, *N*_HC_ = 28) = 222.0, *z* = 1.68, *p* = 0.093, *η*^*2*^ = 0.06, with the AUD patients descriptively showing an Apb and the HCs showing an avoidance bias (Median_AUD_ = 0.305, SD = 2.186; Median_HC_ =− 0.453, SD = 2.027). Boxplots are shown in Fig. 4.

### (Ib’) Bias scores tested against zero within groups to detect true approach versus avoidance biases

In case of previously identified group differences in Apb scores between AUD and HC, bias scores (median Apb) were tested with a Wilcoxon signed rank test against zero within the respective groups, to test whether there were ‘true’ approach/ avoidance biases. For grasping-condition 1, the Apb Median_AUD_ = 1.167 (SD = 1.873) was different from zero at a non-significant trend-level (*t*(13) = 1.85, *p* = 0.064). For the Apb Median_HC_ = − 0.623 (SD = 1.819), it was found that this value did not differ significantly from zero (*t*(13) =  − 0.94, *p* = 0.349).

Concerning grasping-condition 3, the Apb Median_AUD_ = 0.305 (SD = 2.186) was not significantly different from zero (*t*(22) = 1.15, *p* = 0.249). Furthermore, the Apb Median_HC_ = − 0.453 (SD = 2.027) was also not significantly different from zero (*t*(22) =  − 1.30, *p* = 0.194).

### Descriptive PC-AAT data analysis

The median RT_total_ of the raw data including all available RTs (i.e. correctly sorted, no change of direction, alcoholic stimuli; *N* = 4384) for PUSH_alc_ (*n* = 2167 trials available) was Md_RT_total_PUSHalc_PC_ = 919 ms (SD = 439 ms). This led to a 2-SD upper bound of 1797 ms. For PULLing alcoholic stimuli, based on all available RTs (*n* = 2217 trials available) the results were Md_RT_total_PULLalc_PC_ = 869 (SD = 389 ms), corresponding to an upper bound of 1647 ms. Using these upper bounds as filters to sort out RTs that were too long, resulted in a filtered dataset of *N* = 4212 (− 9.6%), with now *n* = 2087 (− 9.6%) available PUSH_alc_ and *n* = 2125 (− 9.6%) available PULL_alc_ (− 9.6%) RTs_total_.

In a next step, PULL_alc_ and PUSH_alc_ RTs were evaluated per participant concerning the availability of a sufficient number of valid RTs (*N* ≥ 25) to compute the respective median scores and standard deviations. Except for two cases in the HC group, sufficient data were available for all cases.

### (II) Inferential PC-AAT data Analysis

**Fig. 5:**
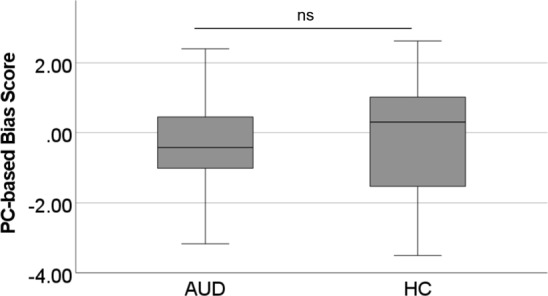
Boxplot for the PC-AAT bias scores per group. *Note*. *ns* non-significant; AUD = alcohol use disorder; HC = healthy controls

The Mann–Whitney U test for independent samples revealed no significant difference in the PC-based Apb scores, *U*(*N*_AUD_ = 25, *N*_HC_ = 30) = 392.0, *z* = 0.287, *p* = 0.774, whereby descriptively, on average, the AUD patients exhibited a tendency for an avoidance bias (Md_AUD_ = − 0.421, SD = 1.368) and HCs a tendency for an Apb (Md_HC_ = 0.305, SD = 1.592). A boxplot can be found in Fig. 5.

### (III) Validity of VR- versus PC-based Bias Scores

All correlations (Spearman rho) between the different bias scores and clinical measures can be found in Table 4. The PC and VR-bias scores generally did not correlate with one another (all *p* > 0.05). Concerning validity of the bias scores, only the VR-based (but not the PC-based) scores showed significant correlations with a clinical criterion, namely impulsivity as measured by the BIS-11, with small to moderate effect size (see Table 4).

**Table 4:** Correlations between different bias scores (PC vs. VR-based) and with clinical variables

Variable		Apb_PC_	Apb_VR_no_grasp_	Apb_VR_grasp_PULLing_
ADS^1^	*r*	− 0.315	− 0.204	− 0.132
	*p*	0.125	0.505	0.558
	*N*	25	13	22
BIS-11	*r*	0.059	0.304 ^†^	0.287^*^
	*p*	0.667	0.096	0.043
	*N*	55	31	50
Apb_VR_no_grasp_	*r*	− 0.103		
	*p*	0.594		
	*N*	29		
Apb_VR_grasp_PULLing_	*r*	0.240	− 0.017	
	*p*	0.100	0.930	
	*N*	48	30	

*p < *.05*

^*†*^*p* < *.10*

## Discussion

The present study was concerned with three major research questions, which will be briefly addressed below. Regarding question (**Ia**), which way of pressing the lever at the underside of the VR-controller (vs. not pressing it), thus mimicking’ grasping’ of the stimuli in VR) was best in terms of feasibility, it was found that grasping condition 3 [grasp PULLing] i.e. pressing the lever to grasp the stimuli while PULLing them towards oneself (while not pressing it when PUSHing stimuli away) was most feasible and superior to the other two grasping-conditions in VR. This was followed by grasping-condition 1 (*never grasp*), while grasping-condition 2 (*always grasp* [i.e. pressing the lever both to ‘grasp’ the stimuli whilst PULLing and PUSHing stimuli]) showed inacceptable rates of correct and valid responses. Concerning (**Ib**) the superiority of different VR-based bias scores (by grasping-condition), only bias scores based on grasping-condition 1 discriminated between patients with AUD and HCs. Furthermore, only grasping-condition 1 revealed a non-significant trend of the median bias score in the AUD group being significantly different from zero. It can thus be concluded, albeit it was only a statistical trend, that using the VR controller *never grasping* might be suited best for detecting the Apb in AUD patients versus HCs. However, given the trend-level concerning AUD and HC differing in bias scores also in the *grasp PULLing* condition, and this condition showing better feasibility than the *never* grasping-condition, it might have potential for further exploration.

Concerning (**II**) the question of similarity versus disparity of findings measuring the bias in VR versus with the classical PC-based version (using a joystick), it was found that the PC-based version was not suited to differentiate between patients with AUD and HCs. Rather an opposite-than-expected result emerged with the AUD patients showing an avoidance bias and the HCs an Apb on average. However, these differences were non-significant, suggesting that the PC-AAT was unable to discriminate between the clinical versus healthy control group. Hence, at this point, it can be concluded that the VR-based AAT is better suited than a matched PC-based version of the AAT to differentiate between AUD patients and HC concerning the Apb.

Finally (**III**), of all bias scores only the VR-based scores for grasping-conditions 1 and 3 showed any evidence of validity, showing small-to-moderate, positive correlations with impulsivity. However, both VR-based bias scores did not correlate with AUD symptom severity. The PC-based bias-score was entirely unrelated to AUD symptom severity and impulsivity. It can be concluded that the VR-based bias assessments in the present study fare better in terms of convergent validity than the PC-based bias assessment, albeit this only applies to observed associations with impulsivity (and not AUD severity).

Although ‘grasping when PULLing only’ (condition 3) was most feasible in executing movements, only a trend-level difference in bias scores between AUD patients and HCs could be observed. ‘Never grasping’ (condition 1) on the other hand best captured an Apb in the AUD group. It is possible that although ‘grasping when PULLing’ represents the most innate and intuitive hand movement and thus descriptively showed highest number of correctly performed trials, the increased cognitive load of carrying out the movement correctly might prolong the RTs and thus prevent a significant Apb from being detected. This issue could be addressed by future studies implementing a ‘VR glove’ enabling the participants to carry out natural hand movements yet taking away the ‘dummy aspect’ of pressing a lever. This way, the concept of embodiment could be implemented even more consequentially, allowing movements to be natural and intuitive rather than artificially coordinated (Dijkstra et al. 2015; Fridland et al. 2017; Dijksterhuis et al. 2001).

### Limitations

The VR-AAT in the present study has been implemented in a simple fashion to ensure comparability with the PC-AAT, as the main goal of this study was to compare both AAT versions and to learn whether Apbs towards alcohol could be detected in the VR-AAT. A drawback of previous alcohol AAT studies had been the lack of comparison to control groups. Undoubtedly, despite the clinical concept, clinical validation should still be a topic of ongoing research. It is therefore important to further validate the effectiveness of bias detection. Although the VR-AAT was set up in a similar setting to the PC-AAT (i.e. sitting on a chair in front of a table, white background, and only 2D pictures appearing) not all aspects could be held constant. While the total duration of the PC-AAT was about 6–10 min, due to its complexity and integration of the different conditions of the VR-AAT, it could take up to 45 min to carry out all trials in the different grasping-conditions. Furthermore, more practice trials were needed to ensure that all three different grasping-conditions were mastered before the main trial started. It can therefore be concluded that the VR-AAT was more time consuming and complex to perform than the original PC-AAT, which may have led to tiring over the course of its implementation. However, all AAT instructions (i.e. pull/push alcohol first) and grasping-conditions were randomized to control for learning effects and tiring. Furthermore, it was randomized whether the PC- or VR-AAT was presented first, and they were never implemented on the same day of assessment. Additionally, as we were interested in assessing approach biases and the questions of interest for people with AUD, patients with other comorbidities (i.e. other SUDs, or severe psychiatric diagnoses such as psychosis or bipolar disorder) were not included in the sample. However, it is important to note that AUD is oftentimes accompanied by other disorders. For example, between 40 and 50% of individuals with AUD also have a lifetime disorder of another substance use disorder (Castillo-Carniglia et al. 2019). Therefore, the results of this study are not generalizable to the entire population of people with AUD, but only to those with a single AUD diagnosis in the absence of other severe psychiatric comorbidities. Lastly, as of now it cannot be said with certainty, what the underlying differences in bias assessment between the PC-AAT and VR-AAT are and further studies should set out to explore this research question.

## Conclusion

This study found that an Apb could be detected in a VR environment for AUD patients, when compared to HCs, but not in a matched PC-AAT version. Additionally, the Apb for AUD was different from zero at a non-significant trend level. (Ia) We found that pressing the lever at the underside of the VR-controller when PULLing stimuli towards the oneself to mimic grasping of stimuli was most feasible compared to other grasping-conditions, potentially because it represents the most intuitive movement. (Ib) However, detecting an Apb and discriminating between AUD patients and HCs was best realized in a ‘*never grasp’* condition, in which participants only performed arm movements in VR without pressing the lever at the underside of the VR-controller. (II) This assessment of the bias was superior to any other assessments, including in the classical PC-setting. (III) Generally, VR-based bias-scores showed better evidence of validity than PC-based bias scores. It can generally be concluded that Apb assessment is both feasible in VR and possibly even superior to PC-based assessments. Hence, further VR-based studies are a promising endeavour. Future studies could focus on enhancing the grasping-component even more, e.g. via using a VR glove, whereby the participant can carry out a natural grasping-movement involving all fingers. Additionally, VRs with typical drinking environments, such as a restaurant-, bar-, or living room setting, could be designed and studied. Furthermore, more realistic or alternative depictions of the alcoholic stimuli (i.e. 3D objects) could be implemented to potentially increase biases.

## Data Availability

We hereby declare that our data and syntaxes (including a documentation of all analyses that were undertaken) are available upon request.
